# Cultivated and Wild Juvenile Thick-Lipped Grey Mullet, *Chelon labrosus*: A Comparison from a Nutritional Point of View

**DOI:** 10.3390/ani11072112

**Published:** 2021-07-15

**Authors:** Jorge García-Márquez, Alba Galafat, Francisco Javier Alarcón, Félix L. Figueroa, Eduardo Martínez-Manzanares, Salvador Arijo, Roberto Teófilo Abdala-Díaz

**Affiliations:** 1 Departamento de Microbiología, Facultad de Ciencias, Campus de Teatinos s/n, Universidad de Málaga, 29071 Málaga, Spain; emmanzanares@uma.es (E.M.-M.); sarijo@uma.es (S.A.); 2 Departamento de Biología y Geología, Campus de Excelencia Internacional CEIMAR, Universidad de Almería, 04120 Almería, Spain; albagalafat@gmail.com (A.G.); falarcon@ual.es (F.J.A.); 3 Departamento de Ecología y Geología, Instituto de Biotecnología y Desarrollo Azul (IBYDA), Campus de Teatinos s/n, Universidad de Málaga, 29071 Málaga, Spain; felix_lopez@uma.es (F.L.F.); abdala@uma.es (R.T.A.-D.)

**Keywords:** amino acids, *Chelon labrosus*, fatty acids, proximate analysis

## Abstract

**Simple Summary:**

The thick-lipped grey mullet (*Chelon labrosus*) has good potential for aquaculture diversification in Europe. However, research studies about this species are scarce, particularly focusing on the nutritional attributes of wild and cultivated thick-lipped grey mullets that might help to optimize its feeding regime. In order to determine the nutritional composition of thick-lipped grey mullet juveniles, wild and cultivated specimens were collected and compared. To do so, the liver fatty acids, muscle proximate composition, fatty acids and amino acids were analyzed. The wild specimens had higher levels of polyunsaturated fatty acids and a higher content of eicosapentaenoic (EPA) and docosahexaenoic (DHA) acids than the farmed specimens. Furthermore, all the amino acid scores were above 100% compared to the Food and Agriculture Organization of the United Nations/World Health Organization (FAO/WHO) standard. This study provides new knowledge and contributes to understanding the nutritional attributes of wild and cultured *C. labrosus* and helps to design diets according to their nutritional demands.

**Abstract:**

The thick-lipped grey mullet (*Chelon labrosus*) is a nominee fish species for aquaculture diversification in Spain because it is an omnivore and euryhaline species, but limited knowledge about the nutritional attributes of this species is available. This study aimed to characterize the chemical composition of wild and cultured fish. The muscle proximate composition, and fatty acid and amino acid profiles were assessed. The cultivated specimens showed a higher lipid content and lower protein and ash contents compared with the wild specimens. The predominant tissue fatty acids in both the wild and cultivated fish were palmitic acid (16:0), oleic acid (18:1n-9) and docosahexaenoic acid (DHA, 22:6n-3). A higher content of arachidonic acid (ARA, 20:4n-6), eicosapentaenoic acid (20:5n-3) and DHA were detected in the muscle of wild mullets, while the fish supplied with commercial pellets showed higher quantities of monounsaturated fatty acids, and lower quantities of saturated fatty acids and polyunsaturated fatty acids (PUFAs). Regarding PUFAs, n-3 fatty acids were predominant in wild mullets, while n-6 and n-9 were more abundant in farmed fish. In terms of amino acid composition, except for histidine in wild specimens, the amino acid amounts were higher than the FAO/WHO standard. In conclusion, *C. labrosus* may contribute to improving the dietary intake of highly polyunsaturated n-3 fatty acids, with a benefit to human health, owing to that fact that a 100-g fillet portion of cultivated and wild *C. labrosus* can provide 770 mg and 1160 mg of EPA and DHA, respectively, which exceeds the 250 mg dietary daily intake recommended by the FAO/WHO.

## 1. Introduction

Aquaculture production has rapidly worldwide expanded, accounting for 52% of fish consumption [1]. This evolution has been caused by the increase in fish consumption, as wild fish catches are no longer sufficient [2]. The low species diversity that contributes to aquaculture production is one of the main causes of its unsustainable development [3,4]. Indeed, although numerous species have been farmed, 85% of the current world fish production relies on about fifteen species [5]. In this sense, the Food and Agriculture Organization of the United Nations (FAO) has recently advocated for diversification concerning species, suggesting that species diversification should be addressed by (i) increasing the number of species cultivated; (ii) increasing the evenness of the cultivated species; and (iii) increasing the diversity within the species currently cultivated through the development of new strains [6,7].

In Europe, one of the major impediments to the growth of aquaculture is that it is based on the production of carnivorous fish [1]. This makes them dependent on fishmeal and fish oil in their diet, which increases their impact on fish stocks. One solution to this problem is the diversification in fish farming, opting for omnivorous or herbivorous species.

Thick-lipped grey mullet (*Chelon labrosus*) has some characteristics that make this species an interesting candidate for aquaculture diversification [8,9]. *C. labrosus* is an omnivorous species in the early stages of development, changing to herbivorous with age [10,11,12]. Similar to other mullets, *C. labrosus* has a high osmoregulatory capacity, allowing it to inhabit a broad range of salinities without affecting its growth rate [9,13]. Recently, Besbes et al. [14] reported that the thick-lipped grey mullet fry production was more efficient (i.e., higher growth rate and better survival rate) in green water than in clear water. Its results demonstrated the possibility to control the production of *C. labrosus* in captivity, although further research is needed to optimize its production.

Fish consumption is recommended because of its high-quality proteins, low cholesterol content and high percentage of n-3 polyunsaturated fatty acids (PUFAs), particularly eicosapentaenoic acid (EPA) and docosahexaenoic acid (DHA), vitamins and minerals [15,16]. Although the protein composition is relatively similar in all fish species, the content of vitamins, fatty acids and micronutrients is more variable [17,18,19].

Recently, the differences in the chemical composition, fatty acid profile and flesh quality between wild and farmed fish species have been reported [20,21,22,23,24]. Several factors, such as species, age, environment (temperature, salinity, etc.), season, the type and availability of foods and diets and feeding regimes, are important factors contributing to variations in the nutritional value of fish. However, as far as we know, there has not been any attempt to study the differences in the proximate composition, fatty acid profile and amino acid content between wild and cultured thick-lipped grey mullet (*C. labrosus*). The aim of this study was, therefore, to investigate these differences in wild and cultured thick-lipped grey mullets. This study can contribute to understanding the nutritional attributes of wild and farmed *C. labrosus* and help to improve the design of diets following their requirements.

## 2. Materials and Methods

### 2.1. Rearing Conditions, Diets and Fish Sampling

Ten thick-lipped grey mullets were collected in March 2019 from a salt marsh of San Fernando, Cádiz (36°28′05″ N 6°10′58″ W, SW Spain), kept alive and transported to the C.I.F.P. Marítimo Zaporito facilities in San Fernando (Cádiz). The fish were reared in 5 m^3^ tanks at the C.I.F.P. Marítimo Zaporito under natural conditions of photoperiod and constant temperature (19–20 °C). Fish were fed for three months with a commercial diet (Tilapia TI-3 feed, Skretting Co., Trouw, France) that contained 32% protein, 6% fat, 3.9% crude fiber, 6% ash, 0.8% total phosphorus, 5000 u kg^−1^ vitamin A and 750 u kg^−1^ vitamin D3. The ingredients of the commercial aquafeed were fishmeal, soybean meal, soybean protein concentrate, processed animal protein from poultry, corn gluten meal, rapeseed oil, wheat meal, vitamins and minerals. The fatty acid profile of the commercial feed is presented in Table 1. Ten wild thick-lipped grey mullet (average weight and length: 60.2 ± 1.6 g and 16.7 ± 1.5 cm, respectively) were caught the same day with a net in a salt marsh of San Fernando, Cádiz (36°28′05″ N 6°10′58″ W, SW Spain), kept alive and transported to the C.I.F.P. Marítimo Zaporito facilities within 20 min. Wild and cultured (average weight and length: 62.5 ± 3.1 g and 17.1 ± 2.0 cm, respectively) animals were euthanized by immersion in water with a 2-phenoxyethanol overdose (1 mL L^−1^). Specimens were then individually weighed, and the muscle and liver were dissected out. Samples were immediately frozen and kept under −80 °C until the subsequent analysis.

### 2.2. Proximate Muscle Composition and Fatty Acid Profile

Proximate analysis of fish muscle samples for dry matter, ash and crude protein (N × 6.25) were determined according to AOAC procedures [25] using elemental analysis (C:N:H) (Fisons EA 1108 analyzer. FisonsInstruments, Waltham, MA, USA). Total lipid content of commercial feed, liver and muscle was performed following the procedure described by Folch [26], using chloroform/methanol (2:1 v/v) as a solvent, and total lipid content was calculated gravimetrically.

Fatty acid profile was determined using gas chromatography according to the method described by Rodríguez-Ruíz et al. [27] (Hewlett Packard, 4890 Series II, Hewlett Packard Company, Avoncale, PA, USA), using a modification of the direct transesterification method described by Lepage and Roy [28].

### 2.3. Indices of Lipid Metabolism and Quality

From the FA profile of fish muscle, different indices were calculated. The peroxidability index (PI) was calculated using the following equation proposed by Arakawa and Sagai [29] (Equation (1)):

PI = (% monoenoic × 0.025) + (% dienoic × 1) + (% trienoic × 2) + (% tetraenoic × 4) + (% pentaenoic × 6) + (% hexaenoic × 8)(1)

The index of atherogenicity (IA) and the index of thrombogenicity (IT) were calculated according to Senso et al. [30] as follows (Equations (2) and (3)):

IA = (12:0 + 4 × 14:0 + 16:0)/[(n-6 + n-3) PUFAs + 18:1 + other MUFAs](2)

IT = (14:0 + 16:0 + 18:0)/[(0.5 × 18:1) + (0.5 × ΣMUFAs) + (0.5 × n-6 PUFAs) + (3 × n-3 PUFAs) + (n-3/n-6)],(3)

where MUFAs and PUFAs stand for monounsaturated fatty acids and polyunsaturated fatty acids, respectively.

The value of flesh-lipid quality (FLQ) indicates the ratio between the sum of eicosapentaenoic acid (EPA, 20:5n-3) and docosahexaenoic acid (DHA, 22:6n-3), and total lipids, expressed as mg per 100 g of edible fillet.

### 2.4. Amino Acid Content and Protein Quality Evaluation

For amino acid analysis, muscle samples were hydrolyzed (20 mg in 1 mL HCl 6M) at 110 °C for 24 h under an inert atmosphere (N_2_). After that, 50 μL of the hydrolysate was mixed with 50 μL of 6 M NaOH. Then, 100 μL of internal standard (2.5 mM norleucine) and 800 μL sodium citrate loading buffer (pH 2.2) was added and mixed by vortex for 5 s and then filtered (0.2 μm). A sample (20 μL) of this mixture was analyzed with a Biochrom 30 amino acid analyzer (Biochrom Ltd., Cambridge, UK), according to the manufacturer’s protocol.

Protein quality evaluation was calculated through amino acid score calculated according to the following formula (Equation (4)):

Amino acid score (%) = ((EAA (mg g protein^−1^)/EAA in reference pattern (mg g protein^−1^)) × 100, (4)

where EAA is the essential AA.

The maintenance amino acid pattern suggested in reference [31] (Table 3, p. 27) was used for calculation.

### 2.5. Statistical Analysis

Results are reported as means ± SD (n = 10). Normal distribution was checked for all data with the Shapiro–Wilk test, while the homogeneity of the variances was obtained using the Levene test. When necessary, an arcsin transformation was performed. Differences between groups were tested using Student’s *t*-test. In all statistical tests used, *p* < 0.05 was considered significantly different. All analyses were performed with SPSS Statistics 25 software (SPSS Inc., IBM Company, Armonk, NY, USA).

## 3. Results

### 3.1. Muscle Proximate Composition

The muscle proximate composition analyses of the wild and cultured thick-lipped grey mullets are presented in Table 2. The wild thick-lipped grey mullets contained significantly higher levels of protein and ash than the cultured mullets. In contrast, the total lipid content was significantly lower in the wild than in the cultured mullets.

### 3.2. Liver Fatty Acids

The liver fatty acid composition of the cultured and wild thick-lipped grey mullets is presented in Table 3. The monosaturated fatty acids (MUFA) and the total n-9 fatty acids proportions were significantly higher in the cultured specimens, mainly due to the higher values of palmitoleic (16:1) and oleic acid (18:1n-9) observed in this fraction. The arachidonic acid (ARA, 20:4n-6), eicosapentaenoic acid (20:5n-3) and docosahexaenoic acid (DHA, 22:5n-3) contents were significantly higher in the wild fish. As a result, although the total n-6 fatty acids tended to increase in the wild fish, significant differences were only observed in the total PUFA and the total n-3 of the wild specimens.

### 3.3. Muscle Fatty Acids

Polyunsaturated fatty acids (PUFA) were the predominant fatty acids in the muscle of the wild specimens (Table 4), while saturated fatty acids (SFA) and monounsaturated fatty acids were significantly higher in the cultured fish. Considered individually, palmitic acid (16:0) was prevailing in the cultured fish (27.3%), followed by oleic acid (18:1n-9; 18.4%) and docosahexaenoic acid (DHA, 22:6n-3; 12%). On the other hand, the wild specimens yielded DHA and eicosapentaenoic acid (EPA, 20:5n-3) as the most abundant fatty acid in muscle (29.8% and 29.4%, respectively), followed by palmitic acid (12.8%). Both DHA and EPA were significantly higher in the wild specimens, thus contributing to a significant increase in the total n-3 content and the n-3/n-6 ratio. As a result of these changes, the wild specimens showed a significantly higher peroxidability index, lipid quality index (FLQ), as well as a lower index of atherogenicity (IA) and thrombogenicity (IT).

**Figure 1:**
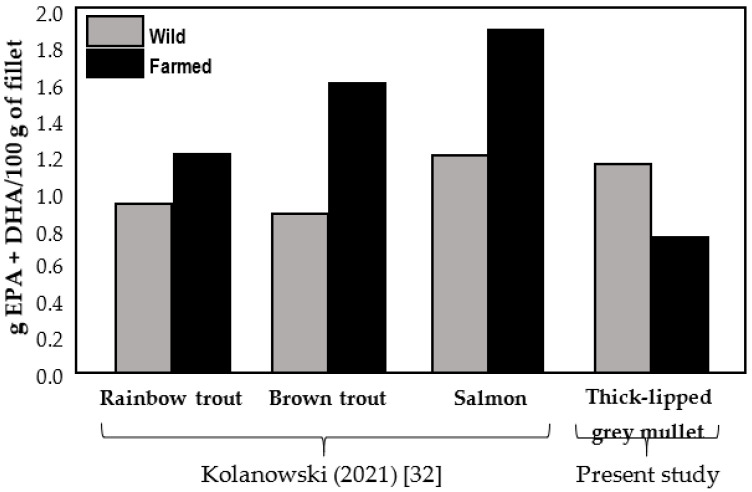
Comparison of content of n-3 long chain PUFAs (g/100 g of raw meat) in wild and cultivated salmonid fish (modified from [32]) and thick-lipped grey mullet fillets (present study).

The content of EPA and DHA in fillets of the wild and cultivated thick-lipped grey mullet was compared with reference values published for salmonids (Figure 1). Thick-lipped grey mullet showed values higher in the muscle of the wild fish than in the cultivated ones. In the case of salmonids, the opposite was reported. Anyway, the n-3 long-chain PUFA content in the fillet of thick-lipped grey mullet was high and nutritionally desirable with content within the range reported for salmonids.

### 3.4. Amino Acid Content and Score

The AA profiles of the wild and cultivated thick-lipped grey mullets are shown in Table 5. In both the wild and cultivated mullets, lysine was the essential AA (EAA) with the highest percentages, followed by leucine. On the other hand, histidine and methionine had the lowest relative content of all the EAAs. The total essential AA was similar in the wild and cultivated mullets. Regarding the non-essential AAs (NEAAs), glutamic and aspartic acid were the AAs with the highest percentages, whereas cysteine had the lowest percentage (below 1%) in both the wild and cultivated mullets. The AA profile of the wild and cultivated mullets showed a significant difference observed in the histidine amount, which was higher in the farmed fish. The content of lysine, serine and glutamic acid was higher in the wild specimens. The EAA/NEAA ratio was statistically higher in the cultivated fish.

In terms of protein quality, the amino acid (AA) with the highest content in both the cultivated and wild thick-lipped grey mullet was lysine (90.3 and 90.3 mg g protein^−1^, respectively), followed by leucine (70.7 and 66.8 mg g protein^−1^ for the cultivated and wild mullets, respectively) (Table 6). Histidine was the AA with the lowest concentration. Except for the content of histidine in the wild specimens, all the AAs from the cultivated and wild thick-lipped grey mullet showed higher concentrations compared to the FAO/WHO reference standard [31] concentration regarding human AA requirements. For that reason, all the AAs showed a score higher than 100% (85% for histidine in the wild mullets; Table 6).

## 4. Discussion

Intensive aquaculture production has raised concerns about the nutritional quality of farmed fish compared to wild fish. Therefore, it is of great importance to evaluate the differences in the quality of wild and farmed fish.

The wild thick-lipped grey mullets had higher crude protein and lower crude lipids than the farmed mullets. This is a common phenomenon observed in the literature when comparing farmed and wild species [21,23,33,34,35,36]. The higher lipid content in the farmed fish could be related to a variety of internal and external factors such as the species of study [37], physiological status [38], season and temperature [39], the availability, dose and the type of feed [40], higher energy consumption by the farmed fish compared with the wild fish [41] and the reduced activity of the cultured fish [42]. In this study, the farmed fish were fed with commercial feed rich in proteins and lipids (32% and 6%, respectively), which could have promoted the increase in lipid content. The high accumulation of lipids in the farmed specimens seems to indicate that these fish preferentially use dietary protein for energy, while the fat extracted from feed is stored in the body [34].

Artificial diets provide a wide range of nutrients to farmed fish, which not only determine the growth rate of the fish, but also the composition of the flesh, in particular the lipid content, which can be modified quantitatively and qualitatively [43]. According to Fountoulaki et al. [44], the dietary fatty acid composition is reflected in the fatty acid composition of marine fish tissues. As expected, palmitic acid (16:0) and oleic acid (18:1n-9) were the primary saturated and monounsaturated fatty acids, respectively, for both the cultured and wild thick-lipped grey mullets. Similar results for other fish species have also been reported in the literature, including other mullet species [45,46]. The higher amount of these acids in fish tissues seems to arise from its high level in commercial feed (Table 1).

The total PUFA relative content was higher in the wild mullets. Among the n-6 series of the fatty acids, the liver and muscle of the cultured specimens had a higher level of linoleic acid (18:2n-6) and a lower content of arachidonic acid (20:4n-6, ARA) than the wild fish. The high amount of linoleic acid in aquafeed (22.1%) is reflected in the fish tissue, although its reduction seems to be related to a conversion of linoleic acid to ARA. Within n-3 PUFA, the percentage of EPA and DHA in the wild thick-lipped grey mullet was three times higher than that found in the cultured fish (59.34% and 19.58%, respectively). High levels of ARA, EPA and DHA in wild fish have been observed in other fish species [33,45,47,48]. These fatty acids play a key role in cellular membrane structure and function, and their dietary imbalance leads to reduced growth and increased fish mortality as well as other pathologies such as liver or intestinal steatosis [49].

The high levels of PUFA, and in particular of n-3 PUFA, in the wild thick-lipped grey mullets could be explained by the natural feeding of these specimens in the salt marsh of San Fernando, which is based on benthic diatoms and epiphytic algae [50,51]. The potential use of microalgae in aquafeeds for aquaculture has been extensively reviewed [52,53]. Overall, their chemical composition is characterized by a considerable protein content (up to 40% protein on a dry matter basis) and an adequate fatty acid profile with substantial amounts of linolenic acid (18:3n-3) and a high proportion of EPA and DHA [54].

The overall difference of fatty acids in muscle is particularly interesting from the point of view of human nutrition. In this sense, EPA and DHA were 50% higher in the muscle of the wild fish than the cultured fish (1.16 g/100 g of fresh fillet vs. 0.77 g/100 g of fresh fillet, respectively). The content of EPA and DHA in fillets of the wild thick-lipped grey mullets was in line with published data for reference fish species (Figure 1). Conversely, the levels of EPA and DHA were lower than those of reference species. This could be due to the different concentration of lipids in the feeds. The lipid content in salmonids’ feeds can reach up to 30%, while the lipid content of the commercial diet provided to the farmed mullets was 6% (Table 1). An alternative to increase the EPA and DHA content in farmed mullets could be the administration of feeds with a higher percentage of lipids and/or with a higher content of EPA and DHA to try to match the values to the levels of the reference species. Indeed, the higher content of EPA and DHA in wild mullets is also reflected in the higher levels of the fish lipid quality index (FLQ), the peroxidability index and the n-3/n-6 ratio in the wild specimens, as well as lower indices of atherogenicity (IA) and thrombogenicity (IT), showing that the marine environment provides an excellent source of n-3 rich foods. There is compelling evidence that the n-3 PUFA, particularly EPA and DHA, and an adequate balance of n-6/n-3 fatty acids in human diets play a determinant role in the prevention of many diseases, such as obesity, Alzheimer’s and brain disorders [55,56,57,58]. For this reason, the Food and Agriculture Organization (FAO), the World Health Organization (WHO) and the European Food Safety Authority (EFSA) recommend a daily EPA and DHA intake of at least 250 mg [59,60]. Our results were higher than the given reference value for good health, indicating that cultivated and wild individuals can be considered as a good source of EPA and DHA in human diets.

Amino acid composition influences the nutritional quality of proteins, especially the essential amino acid pattern [61]. Moreover, the amino acid pattern is a significant nutritional particularization for the development of feed for a new candidate fish species for the aquaculture industry. To the best of our knowledge thus far, the present study is the first attempt to investigate the amino acid composition of cultured and wild thick-lipped grey mullets.

In the present study, the highest levels of the essential amino, acids lysine, leucine, arginine and valine, and the non-essential amino acids, alanine and glutamic and aspartic acid, were recorded in the muscle tissue of wild and cultured thick-lipped grey mullets, with only significant differences in the histidine, serine and glutamic acid content. Histidine is a precursor of histamine, and it is required for various roles in protein interaction and the growth and repair of tissue [62]. Glutamic acid plays an important role in amino acid metabolism, including transamination reactions and the synthesis of key molecules [63]. Serine is the precursor of glycine, cysteine and tryptophan and plays an important role in cell signaling and gluconeogenesis [64]. Similar amino acid profiles have been described in farmed versus wild-caught specimens [21,34,35]. In general, lysine, leucine and arginine are considered to be the most abundant EAAs in aquatic animals [65].

In terms of total essential amino acids (ΣEAA) and total non-essential amino acids (ΣNEAA), our results agree with earlier reports [21,66]. The ratio of total essential amino acids to total non-essential amino acids (ΣEAA/ΣNEAA ratio) was statistically higher in the cultivated than in the wild specimens. Nevertheless, the ratio of both groups (wild and cultivated) was higher than the reference value of >60% provided by the FAO/WHO [67], indicating that cultivated and wild specimens can be considered as a food source with high-quality protein. Furthermore, concerning the reference amino acid contents of the FAO/WHO [31], all the AAs in the present study showed higher concentrations compared to the FAO/WHO reference standard concentration regarding AA requirements, except for histidine in the wild specimens, showing that amino acids were present at sufficient levels.

## 5. Conclusions

The data presented in this work reveal the differences in fatty acid composition and amino acid profile between cultured and wild thick-lipped grey mullets and indicate the nutritional superiority of wild fish. The lower proportion of n-3 PUFA in farmed fish may reduce the nutritional quality of its lipid components, though one serving (130 g) of fillets might provide a significant dose of EPA and DHA up to 0.99 g. In this sense, the aquaculture industry should aim towards an increase in the total n-3 PUFA content of cultured thick-lipped grey mullets choosing the proper dietary lipid intake, considering the supplementation with microalgae due to its good fatty acid profile (for instance *Nannochloropsis gaditana* as a source of EPA, and *Schizochytrium* sp. as a source of DHA), which would allow for tailoring the fatty acid composition of farmed fish to meet both health benefits and consumer requirements.

## Figures and Tables

**Table 1:** Fatty acid profile (% of total fatty acids) of the commercial diet.

Fatty Acids	%
14:0	2.5
16:0	21.3
18:0	4.7
16:1	3.0
18:1n-9	18.6
20:1n-9	1.8
18:2n-6	22.1
20:4n-6, ARA	0.9
18:3n-3	2.3
20:5n-3, EPA	5.7
22:5n-3	1.3
22:6n-3, DHA	14.2
ƩSFA	28.5
ƩMUFA	23.3
ƩPUFA	46.5
Other FA	1.7
Ʃn-3	23.5
Ʃn-6	23
n-3/n-6	1.02

SFA: saturated fatty acids; MUFA: monounsaturated fatty acids; PUFA: polyunsaturated fatty acids; ARA: arachidonic acid; EPA: eicosapentaenoic acid; DHA: docosahexaenoic acid.

**Table 2:** Muscle proximate composition (% wet weight) of wild and cultivated thick-lipped grey mullets.

	Cultivated	Wild	*p*
Total protein	20.16 ± 0.02	22.06 ± 0.01 *	<0.001
Tota lipid	3.91 ± 0.66 *	1.96 ± 0.17	0.007
Ash	1.38 ± 0.00	1.52 ± 0.00 *	<0.001
Moisture	73.01 ± 0.41	72.89 ± 0.91	n.s.

Values are expressed as average ± SD (n = 10 fish per group). Asterisks denote significant differences (*p* < 0.05). n.s.: not significant.

**Table 3:** Liver fatty acid profile (% of total fatty acids) of wild and cultivated thick-lipped grey mullets.

Fatty Acids	Cultivated	Wild	*p*
14:0	12.60 ± 3.71	25.74 ± 1.05 *	0.040
16:0	25.41 ± 1.34 *	10.84 ± 0.06	0.003
18:0	5.27 ± 0.11 *	2.26 ± 0.00	0.013
16:1	7.56 ± 0.53 *	1.25 ± 0.07	0.003
18:1n-9	20.65 ± 1.90 *	8.22 ± 0.24	0.011
18:2n-6	4.58 ± 0.54 *	2.98 ± 0.01	0.023
18:3n-3	0.69 ± 0.12	0.81 ± 0.04	n.s.
20:1n-9	0.03 ± 0.00	2.77 ± 0.38 *	0.030
20:4n-6, ARA	0.39 ± 0.09	3.26 ± 0.06 *	0.018
20:4n-3	0.85 ± 0.06	1.50 ± 0.35	n.s.
20:5n-3, EPA	1.86 ± 0.12	10.36 ± 0.09 *	<0.001
22:5n-3	4.73 ± 0.13 *	2.64 ± 0.09	0.002
22:6n-3, DHA	8.48 ± 0.53	15.49 ± 0.07 *	0.004
ƩSFA	43.27 ± 2.48	38.85 ± 1.11	n.s.
ƩMUFA	32.15 ± 2.75 *	14.14 ± 0.34	0.011
ƩPUFA	21.58 ± 0.48	33.24 ± 0.67 *	0.002
Other FA	2.99 ± 0.76	12.74 ± 0.43 *	0.004
Ʃn-3	16.61 ± 0.60	31.03 ± 0.56 *	0.001
Ʃn-6	4.97 ± 1.09	6.24 ± 0.06	n.s.
Ʃn-9	20.68 ± 1.90 *	10.99 ± 0.14	0.018
n-3/n-6	3.44 ± 0.87	4.97 ± 0.04	n.s.
EPA/DHA	0.22 ± 0.00	0.67 ± 0.00 *	<0.001

Values are expressed as average ± SD (n = 10 fish per group). Asterisks denote significant differences (*p* < 0.05). SFA: saturated fatty acids; MUFA: monounsaturated fatty acids; PUFA: polyunsaturated fatty acids; ARA: arachidonic acid; EPA: eicosapentaenoic acid; DHA: docosahexaenoic acid; n.s.: not significant.

**Table 4:** Muscle fatty acid profile (% of total fatty acids) of wild and cultivated thick-lipped grey mullets.

Fatty Acids	Cultivated	Wild	*p*
14:0	3.59 ± 0.17 *	0.91 ± 0.19	<0.001
16:0	27.34 ± 1.93 *	12.86 ± 3.12	0.002
18:0	8.43 ± 1.30	6.39 ± 2.34	n.s.
16:1	5.86 ± 0.96 *	2.59 ± 0.87	0.011
18:1n-9	18.40 ± 2.86 *	3.62 ± 0.46	<0.001
18:2n-6	7.04 ± 2.73 *	1.41 ± 0.88	0.027
18:3n-6	0.24 ± 0.05	0.46 ± 0.22	n.s.
18:3n-3	0.81 ± 0.10	0.90 ± 0.46	n.s.
20:1	1.24 ± 0.15 *	0.14 ± 0.04	<0.001
20:3n-6	0.27 ± 0.05	0.72 ± 0.24 *	0.030
20:4n-6, ARA	2.83 ± 0.59	6.12 ± 1.89 *	0.044
20:3n-3	0.05 ± 0.01	0.12 ± 0.05	n.s.
20:5n-3, EPA	7.53 ± 2.03	29.46 ± 3.91 *	0.002
22:6n-3, DHA	12.05 ± 3.55	29.88 ± 4.15 *	0.015
Total lipids (mg g^−1^ d.w.)	144.87 ± 1.48 *	72.30 ± 0.74	<0.001
Other FA	3.22 ± 0.78	4.41± 1.15	n.s.
ƩSFA	39.36 ± 3.31 *	20.16 ± 5.49	0.019
ƩMUFA	25.50 ± 3.91 *	6.35 ± 1.03	0.001
ƩPUFA	30.80 ± 7.83	72.69 ± 6.87 *	0.015
Ʃn-3	20.41 ± 9.74	60.36 ± 3.85 *	0.002
Ʃn-6	10.39 ± 2.23	8.71 ± 3.05	n.s.
Ʃn-9	18.40 ± 2.86 *	3.62 ± 0.46	<0.001
n-3/n-6	2.17 ± 1.55	7.41 ± 2.19 *	0.027
EPA/DHA	0.62 ± 0.05	1.02 ± 0.31	n.s.
PI ^1^	163.67 ± 69.14	447.07 ± 34.62 *	0.003
IT ^2^	0.46 ± 0.15 *	0.10 ± 0.03	0.016
IA ^3^	0.74 ± 0.10 *	0.22 ± 0.07	0.001
FLQ ^4^	14.01 ± 1.72	57.51 ± 1.57 *	0.002

Values are expressed as average ± SD (n = 10 fish group). Asterisks denote significant differences (*p* < 0.05). SFA: saturated fatty acids; MUFA: monounsaturated fatty acids; PUFA: polyunsaturated fatty acids; ARA: arachidonic acid; EPA: eicosapentaenoic acid; DHA: docosahexaenoic acid; n.s.: not significant. ^1^ PI = (% monoenoic × 0.025) + (% dienoic × 1) + (% trienoic × 2) + (% tetraenoic × 4) + (% pentaenoic × 6) + (% hexaenoic × 8); ^2^ IT = (14:0 + 16:0 + 18:0)/((0.5 × 18:1) + (0.5 × ΣMUFAs) + (0.5 × n-6 PUFAs) + (3 × n-3 PUFAs) + (n-3/n-6)); ^3^ IA = (12:0 + 4 × 14:0 + 16:0)/((n-6 + n-3) PUFAs + 18:1 + other MUFAs); ^4^ FLQ (%) = ((20:5n-3 + 22:6n-3)/total lipid) × 100.

**Table 5:** Muscle amino acid profile (g 100 g muscle^−1^ DW) of wild and cultivated thick-lipped grey mullets.

Amino Acids	Cultivated	Wild	*p*
Essential Amino Acids (EAAs)			
Valine	3.45 ± 0.02	3.31 ± 0.12	n.s.
Methionine	1.84 ± 0.01	1.91 ± 0.03	n.s.
Isoleucine	3.06 ± 0.03	2.97 ± 0.10	n.s.
Leucine	5.28 ± 0.01	5.46 ± 0.16	n.s.
Threonine	3.01 ± 0.01	3.11 ± 0.08	n.s.
Phenylalanine	2.38 ± 0.05	2.44 ± 0.02	n.s.
Histidine	1.26 ± 0.01 *	1.04 ± 0.02	0.006
Lysine	6.74 ± 0.00	7.38 ± 0.18	n.s.
Arginine	3.99 ± 0.01	4.19 ± 0.11	n.s.
ƩEAA	29.17 ± 0.13	29.90 ± 0.80	n.s.
Non-essential amino acids (NEAAs)			
Aspartic acid	6.95 ± 0.00	7.33 ± 0.16	n.s.
Tyrosine	2.09 ± 0.07	2.25 ± 0.03	n.s.
Serine	2.71 ± 0.01	2.95 ± 0.04 *	0.011
Glutamic acid	9.89 ± 0.06	10.66 ± 0.24 *	0.048
Glycine	2.85 ± 0.01	2.99 ± 0.09	n.s.
Alanine	5.13 ± 0.00	5.28 ± 0.13	n.s.
Cysteine	0.21 ± 0.03	0.19 ± 0.01	n.s.
Proline	4.29 ± 0.54	4.09 ± 0.24	n.s.
ƩNEAA	35.96 ± 0.63	37.66 ± 0.97	n.s.
Ratio EAA/NEAA	0.81 ± 0.01 *	0.79 ± 0.00	0.014

Values are expressed as average ± SD (n = 10 fish per group). Asterisks denote significant differences (*p* < 0.05). NEAA: Non-essential amino acids; EAA: essential amino acids, marked in the first column with asterisks. n.s.: not significant.

**Table 6:** Essential amino acid content (mg g protein^−1^) of wild and cultivated thick-lipped grey mullets compared to the FAO/WHO reference standard (mg g protein^−1^) and AA scores (%).

Amino Acids	mg g Protein^−1^			^1^ AA Score (%)	
	Cultivated	Wild	FAO/WHO Standard	Cultivated	Wild
Valine	46.2	40.5	39	118	104
Isoleucine	41.0	36.3	30	137	121
Leucine	70.7	66.8	59	120	113
Threonine	40.3	38.1	23	175	165
Phenylalanine + Tyrosine	59.9	57.4	38	158	151
Histidine	16.9	12.7	15	112	85
Methionine + Cysteine	27.5	25.7	22	125	117
Lysine	90.3	90.3	45	201	201

^1^ AA Scorse (Amino acid score) (%) = [(EAA (mg g protein^−1^)/EAA in reference pattern (mg g protein^−1^)) × 100]; EAA = Essential AA.

## Data Availability

Data presented in this study are available from the corresponding authors upon reasonable request.
